# Large Deletion of MAGT1 Gene in a Patient with Classic Kaposi Sarcoma, CD4 Lymphopenia, and EBV Infection

**DOI:** 10.1007/s10875-016-0341-y

**Published:** 2016-10-21

**Authors:** Immacolata Brigida, Maria Chiriaco, Silvia Di Cesare, Davide Cittaro, Gigliola Di Matteo, Stefania Giannelli, Dejan Lazarevic, Matteo Zoccolillo, Elia Stupka, Alessandro Jenkner, Paola Francalanci, Susanna Livadiotti, Aaron Morawski, Juan Ravell, Michael Lenardo, Caterina Cancrini, Alessandro Aiuti, Andrea Finocchi

**Affiliations:** 1 San Raffaele Telethon Institute for Gene Therapy (SR-TIGET), San Raffaele Scientific Institute, Via Olgettina, 60, 20132 Milan, Italy; 2 Department of Systems Medicine, University of Rome Tor Vergata, Rome, Italy; 3 Division of Immunology and Infectious Diseases Department of Pediatrics, Bambino Gesù Children Hospital, Rome, Italy; 4 Center for Translational Genomics and Bioinformatics, Hospital San Raffaele, Milan, Italy; 5 Department of Pathology, Bambino Gesù Children Hospital, Rome, Italy; 6 Molecular Development of the Immune System Section, NIAID, Bethesda, MD USA; 7 Pediatric Immunohematology and Bone Marrow Transplantation Unit, IRCCS San Raffaele Scientific Institute, Milan, Italy; 8 Vita-Salute San Raffaele University, Milan, Italy

**Keywords:** MAGT1 deletion, Sequencing, Kaposi Sarcoma

## To the Editor

Kaposi sarcoma (KS) in children is rare and generally associated with loss of CD4+ T cells in human immunodeficiency virus (HIV) infected patients and impaired immunity to human herpes virus 8 (HHV-8), or is caused by specific single-gene inborn errors in *IFNGR1*, *WAS*, *STIM1*, and *OX40* genes, that apparently confer selective protective immunity against HHV-8 infection in endothelial cells. Four clinical and epidemiological forms of KS have been defined: the endemic (typical of the Sub-Saharan African), the classical (observed in the Mediterranean Basin), the epidemic (mainly associated with HIV co-infection), and the iatrogenic (due to transplantation-related immunosuppression). Although a small fraction of affected individuals can develop KS after infection with HHV-8, very few reports described a direct relationship between immunodeficiency and clinical spectrum of KS [1–3]. Classical KS is rare in children, although several reports associated its development with inborn errors of immunodeficiency.

The hallmark of X-linked immunodeficiency with magnesium defect, Epstein–Barr virus infection (EBV), and neoplasia (X-MEN) disease is uncontrolled EBV infection, causing a strong susceptibility to B cell lymphoma, even in childhood [4]. To date, 10 male X-MEN patients have been described [5] with CD4-lymphopenia and susceptibility to EBV infection ([4], S1), but none had shown KS manifestations. Herein, we describe for the first time a possible relationship between KS and a magnesium-specific transporter defect caused by a large deletion in the *MAGT1* gene identified by whole exome (WES) and confirmed by whole genome (WGS) sequencing in a young Italian male patient.

**Fig. 1:**
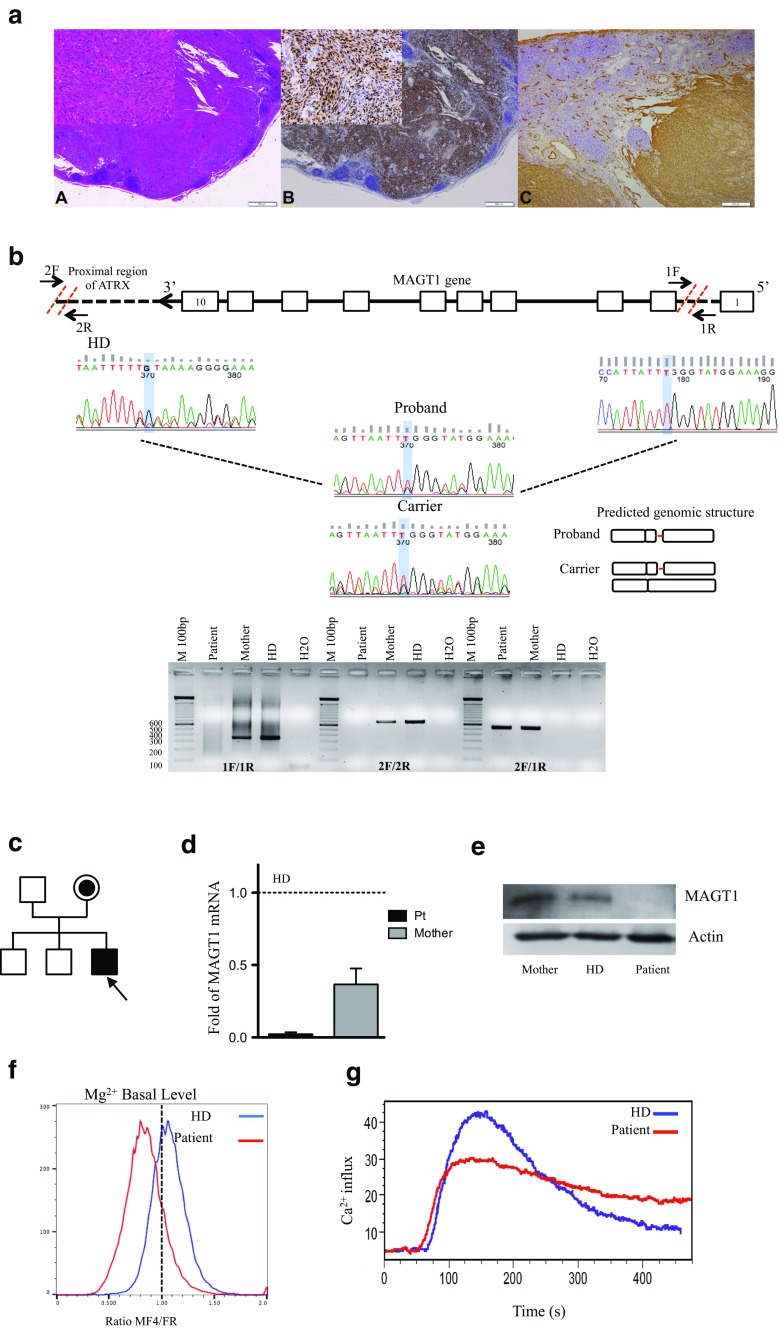
Characterization of X-MEN patient. **a** Lymph node involvement by Kaposi sarcoma: *A* diffuse proliferation of spindle cells forming slits containing red blood cells (HE, 4×, inset 20×), *B* HHV-8 positivity in nuclei of spindle cells (4×, inset 20×), and *C* CD34 positivity of spindle cells (10×). **b** Schematic representation of primers’ design for the identification of the deletion in MAGT1 gene. Deletion in the proband and carrier was confirmed by genomic amplification/DNA sequencing. Predicted structure is showed. **c** Pedigree of the family. The patient is indicated by an *arrow*. **d** Quantitative RT-PCR showing expression of MAGT1 mRNA in T cells normalized to telomerase. Results represented as relative to HD. **e** Western blot on T cells from patient, mother, and HD. MAGT1 30KDa. Actinβ 40 kDa. **f** Mg2+ basal levels in the patient (*red line*) and HD (*blue line*). **g** Calcium flux in freshly isolated PBMC from the patient and HD stimulated with anti-OKT3 (5 μg/mL) shown as percentage of responding cells as a function of time. HD: healthy control

Our patient is an Italian male from the Mediterranean Basin referred to the Ospedale Pediatrico Bambino Gesù at the age of 3 years for cognitive and language delay and a history of recurrent infections. During infancy, recurrent upper respiratory infections and lymphadenopathies were observed and resolved with antibiotic therapy. Respiratory failure caused a subsequent hospitalization at the age of 4 years. At 5 years of age, he developed a cervical lymph node enlargement initially interpreted as a mesenchymal malignancy suggestive of monophasic synovial sarcoma, treated according to the national RMS96 protocol for soft-tissue sarcomas (including ifosfamide, vincristine, and actinomycin D). Review of the original pathology before chemotherapy performed because of unresponsive disease included HE staining (Fig. 1 slice A) and immunohistochemistry for HHV-8 (Fig. 1 slice B) and CD34 (Fig. 1 slice C). Both markers stained positively in spindle cells, thus supporting the histological diagnosis of KS. Unless the clinical presentation is consistent with the endemic form of KS, the patient was negative for HIV antigen and, given his geographic location, was considered as affected by the classic form of KS as previously reported in other known inherited immunodeficiencies [1–3]. Chemotherapy was discontinued, and the patient subsequently received weekly subcutaneous peginterferon alfa-2b (PegIntron) and IVIG every 21 days. This led to a complete remission of disease. Gallbladder stones were detected at 6 years of age. Because of recurrent respiratory infections, bronchiectasis and hypogammaglobulinemia, the patient was referred to our Immunology Unit. Informed consent was obtained from the patient included in the study and was approved by the Institutional Ethical Committee of Ospedale Pediatrico Bambino Gesù and signed by his family. Immunological investigations confirmed defects in various components of adaptive and innate immunity (Table S1). Mild reduced frequency of T cell subsets was observed, with severely decreased naïve CD4 and CD8 T cell counts. B cell compartment progressively expanded, but a marked reduction of memory B cells and almost absent memory and switched memory B cells were noted. Further functional characterization of B cells revealed proliferation after CpG stimulation, but no differentiation to Ig secreting B cells was observed, with undetectable levels of IgG and only small amounts of IgM and IgA in the supernatants. Chronically elevated EBV levels (range 1763–112.000 copies/mL) were confirmed in the absence of a complete seroconversion (no anti-EBNA production).

Supposing a primary immunodeficiency (PID), Sanger sequencing was started for known genes as causative of defects in lymphocytes and infections (i.e., *RAG*, *JAK3*, *CXCR4*), but no mutations were found. Considering the clinical hallmarks of idiopathic CD4 lymphopenia, WES was started and among 113 million reads and a mean coverage of 109X, variants were annotated to (1) dbSNP database, (2) the in-house internal database, and (3) the ESP6500 database. Only variants predicted as probably damaging or damaging (Polyphen2) in either heterozygous or homozygous forms (gMAF <0.05 for rare diseases, SIFT <0.5) were considered. No PID-causative genes were identified by comparing the exome data with the list of PID genes reported in Al-Herz et al. [S2] (Table S2). Although no mutations were observed in KS-associated genes [1–3], two heterozygous variants were found in the APOE and FCGR3A genes. Although no further analyses were performed on these variants to directly correlate these genes with the clinical phenotype of KS, we cannot exclude a genetic predisposition to develop KS in this clinical case.

We then moved to study large structural variants, and by copy number variation (CNV) algorithm [S3], we detected a large deletion in the MAGT1 gene (76 Kb deletion at hg19, chrX:77,056,603–77,142,993, extending from exon 2 to the proximity of the ATRX promoter; Fig. 1b). WGS with low coverage (∼10×) was then applied as confirmatory test in order to identify the correct breakpoint sites. In-depth analysis of the family indicated that the mother was the carrier of the mutation (Fig. 1b, Tables S3–S4 for primers design), while other family members were normal (Fig. 1c). We confirmed the mutation also by quantitative real-time PCR on cDNA (Fig. 1d and Figure S2). As expected, MAGT1 protein was expressed in the mother and healthy donor (HD) (Fig. 1e). Consistently with the diagnosis of X-MEN disease[S4], our patient has reduced basal levels of free Mg^2+^ (Fig. 1f), which impaired proper surface expression of NKG2D, which was also decreased at the intracellular level and in CD8+ T cells (data not shown).

NK cell maturation is associated with the sequential acquisition of specific receptors and functions. CD161, CD56, and the activating receptor NKp44 are the first cell-surface markers expressed during early NK cell development. Other receptors, including NKG2D and CD16, are gradually acquired during later stages of differentiation [S5]. The frequency of NK cells in this patient was reduced for age, with slight impairment in their cytotoxicity against K562. Although CD16+CD56^dim^ was detected, the expression of lineage-committed markers CD161 and CD16 was decreased twofold as compared to controls (Figure S1A–B and data not shown). To our knowledge, this is the first description of an impairment in the early stage of NK cells development in X-MEN patients.

Since abrogation of Mg^2+^ influx due to MAGT1 deficiency has been reported to be associated with decreased Ca^2+^ influx in T cells [S6], we studied Ca^2+^ influx in patient’s PBMC stimulated with OKT3 (5 μg/ml) (Fig. 1g). We found a similar increase in Ca^2+^ influx but the ratio of peak/background was lower in the patient.

An in-depth time course analysis of TCR activation showed a lower pick of phosphorylated PLCγ1 at 5 min after stimulation with H_2_O_2_ (5 mM) in the patient and his mother with respect to HD [S7-S8] (Figure S1C). Moreover, the ability to phosphorylate PLCγ1 was reduced by four- and twofolds in the two samples as compared to the control, and the patient’s cells failed to sustain TCR activation at more than 30 min. Our case report supports the involvement of Mg^2+^ influx in the defective PLCγ1 phosphorylation, which controls Ca^2+^ influx after T cell activation, although we cannot exclude a direct effect of MAGT1 protein on the modulation of T lymphocyte calcium influx.

Susceptibility to severe EBV infection in patients with X-linked lymphoproliferative disease is a classic model of a narrow infectious phenotype, caused by the requirement for a specific co-stimulatory receptor–ligand interaction between T cells and EBV-infected antigen-presenting B cells. The reduction of NKG2D in both NK^+^ and CD8^+^ cells debilitates specific NKG2D ligand-mediated cytotoxic functions, which play essential roles in anti-EBV and antitumor immunity.

This case describes for the first time the association of KS with MAGT1 deficiency in a pediatric patient with clinical presentation atypical from known pediatric and adult classic KS. KS in childhood, whether isolated or associated with other infections, should be investigated for rare single-gene inborn errors of immunity in the absence of HIV infection. In addition to the improvement of antiviral immunity, control of EBV infection and avoidance of lymphoma, free intracellular Mg^2+^ may also play an essential role in the control of HHV8 infection. Our study provides evidence that the combination of novel techniques (WES and WGS) in a single patient can lead to the successful identification of the genetic basis of rare monogenic immunodeficiency, expanding the clinical variability associated with MAGT1 mutations.

## Electronic supplementary material

Below is the link to the electronic supplementary material.

ESM 1 (EPS 1261 kb)

ESM 2 (EPS 1862 kb)

ESM 3 (DOCX 53 kb)

## Abbreviations

EBNA: Epstein–Barr nuclear antigen
EBV: Epstein–Barr virus
HE: Hematoxylin eosin
HHV-8: Human herpes virus 8
HIV: Human immunodeficiency virus
IVIG: Intravenous immunoglobulin
KS: Kaposi Sarcoma
MFI: Mean fluorescence intensity
PBMC: Peripheral blood mononuclear cells
PID: Primary immunodeficiency
PLCγ1: Phospholipase C gamma 1
TCR: T cell receptor
WES: Whole exome sequencing
WGS: Whole genome sequencing
X-MEN: X-linked immunodeficiency with magnesium defect, Epstein–Barr virus infection, and neoplasia
