# Supplementary material for: Large Deletion of MAGT1 Gene in a Patient with Classic Kaposi Sarcoma, CD4 Lymphopenia, and EBV Infection
Source: J Clin Immunol. 2016 Oct 21;37(1):32–5. doi: 10.1007/s10875-016-0341-y (PMC5226982; doi:10.1007/s10875-016-0341-y)
Supplement: Supplementary file 3 — (DOCX 53 kb) [file 10875_2016_341_MOESM3_ESM.docx]

**Large deletion of MAGT1 gene in a patient with classic Kaposi sarcoma, CD4 lymphopenia and EBV infection.**

Immacolata Brigida^*^ PhD^1^, Maria Chiriaco^*^ PhD^2^, Silvia di Cesare BSc^3^, Davide Cittaro PhD^4^, Gigliola Di Matteo BSc^2^, Stefania Giannelli PhD^1^, Dejan Lazarevic MD^4^, Matteo Zoccolillo MSc^1^, Elia Stupka PhD^4^, Alessandro Jenkner MD^3^, Paola Francalanci MD^5^, Susanna Livadiotti MD^3^, Aaron Morawski MSc^6^, Juan Ravell MD^6^, Michael J Lenardo MD^6^, Caterina Cancrini MD PhD^2,3^, Alessandro Aiuti^†‡^ MD PhD^1,6,7^ and Andrea Finocchi^†‡^ MD PhD^2,3^.

**Affiliations**

^1^San Raffaele Telethon Institute for Gene Therapy (SR-TIGET), IRCCS San Raffaele Scientific Institute, Milan, Italy; ^2^Department of Systems Medicine, “University of Rome Tor Vergata", Rome, Italy; ^3^Division of Immunology and Infectious Diseases Department of Pediatrics, Bambino Gesù Children Hospital, Rome, Italy; ^4^Center for Translational Genomics and BioInformatics - Hospital San Raffaele, IRCCS San Raffaele Scientific Institute, Milan, Italy; ^5^Division of Immunology and Infectious Diseases Department of Pathology, Bambino Gesù Children Hospital, Rome, Italy; ^6^Molecular Development of the Immune System Section, NIAID, Bethesda MD; ^6^Pediatric Immunohematology and Bone Marrow Transplantation Unit, IRCCS San Raffaele Scientific Institute, Milan, Italy; ^7^Vita-Salute San Raffaele University, Milan, Italy.

* IB and MC contributed equally to this work

^†^ AA and AF shared last authorship

**Address corresponding author**

^‡^ Alessandro Aiuti, MD, PhD, San Raffaele Telethon Institute for Gene Therapy (SR-TIGET), Pediatric Immunohematology and Bone Marrow Transplantation Unit, San Raffaele Scientific Institute, Via Olgettina, 60; 20123 Milano; Tel.: +39 02 26436564; fax: +39 02 26436545; e-mail: [aiuti.alessandro@hsr.it](mailto:aiuti.alessandro@hsr.it)

^‡^ Andrea Finocchi, MD, PhD, DPUO-Childrens’ Hospital Bambino Gesù and University of Rome Tor Vergata. Rome, Italy, Tel: +39 06 68592508; Fax: +39 06 68592508; e-mail: [andrea.finocchi@uniroma2.it](mailto:andrea.finocchi@uniroma2.it)

**Supplementary Bibliography**

S1. Dhalla F, Murray S, Sadler R, Chaigne-delalande B, Sadaoka T, Soilleux E, et al. Identification of a Novel Mutation in MAGT1 and Progressive Multifocal Leucoencephalopathy in a 58-Year-Old Man with XMEN Disease. J Clin Immunol. 2015;35:112–8.

S2. Al-Herz W, Bousfiha A, Casanova JL, Chapel H CM, Cunningham-Rundles C, Etzioni A, Fischer A, Franco JL GR, Hammarström L, Nonoyama S, Notarangelo LD, Ochs HD, Puck JM, Roifman CM SR and TM. Primary immunodeficiency diseases:an update on the classification from the International Union of Immunological Societies Expert Committee for Primary Immunodeficiency. Front. Immunol. 2011;2:1–26.

S3. Miyatake S, Koshimizu E, Fujita A, Fukai R, Imagawa E, Ohba C, et al. Detecting copy-number variations in whole-exome sequencing data using the eXome Hidden Markov Model: an “exome-first” approach. J. Hum. Genet.. Nature Publishing Group; 2015;175–82.

S4. Chaigne-Delalande B, Li F-Y, O’Connor GM, Lukacs MJ, Jiang P, Zheng L, et al. Mg2+ regulates cytotoxic functions of NK and CD8 T cells in chronic EBV infection through NKG2D. Science (80) 2013;341:186–91.

S5. Montaldo E, Vitale C, Cottalasso F, Conte R, Glatzer T, Ambrosini P, et al. Human NK cells at early stages of differentiation produce CXCL8 and express CD161 molecule that functions as an activating receptor. Blood. 2012;119:3987–96.

S6. Li F, Lenardo M, Chaigne-Delalande B. Loss of MAGT1 abrogates the Mg 2+ flux required for T cell signaling and leads to a novel human primary immunodeficiency. Magnes. Res. 2011;24.

S7. Simeoni L, Bogeski I. Redox regulation of T-cell receptor signaling. Biol. Chem. 2015. p. 555–68.

S8. Knyazhitsky M, Moas E, Shaginov E, Luria A, Braiman A. Vav1 oncogenic mutation inhibits T cell receptor-induced calcium mobilization through inhibition of phospholipase Cγ1 activation. J. Biol. Chem. 2012;287:19725–35.

S9. Shearer WT, Rosenblatt HM, Gelman RS, Oymopito R, Plaeger S, Stiehm ER, et al. Lymphocyte subsets in healthy children from birth through 18 years of age: The pediatric AIDS clinical trials group P1009 study. J Allergy Clin Immunol. 2003;112(5):973–80.

S10. Burgio GR, Perinotto G UA. Pediatria Essenziale. UTET, editor. 1991.

S11. Duchamp M, Sterlin D, Diabate A, Uring-Lambert B, Guérin-El Khourouj V, Le Mauff B, et al. B-cell subpopulations in children: National reference values. Immunity, Inflamm Dis. 2014;2(3):131–40.

**Supplementary Figure legends**

**Figure S1. Phenotypic and functional characterization of XMEN patient.** (A) NK cell development and expression of surface markers in Patient and HD. (B) MFI (mean fluorescence intensity) of NKG2D and CD161. Unpaired *t*-test, **** p<0.001. (C) PLCγ1 phosphorylation in T cells from HD, mother and patient, stimulated with H_2_O_2_ at different time points. Shaded areas represent fold decrease during time of acquisitions.

**Figure S2. cDNA analysis of MAGT1.** (A) Primer design for cDNA amplification. (B) Amplification of cDNA in the patient and the family in comparison with an unrelated HD. No amplification was obtained in patient’s cDNA. GAPDH is used as internal control.

**Table S1. Phenotypic and functional characterization of XMEN patient.**

|  | Pt  (5-years-old) | Normal values for age [S9] | Pt (7-years-old) | Normal values for age [S9] | Pt (13-years-old) | Normal values for age [S9] |
| --- | --- | --- | --- | --- | --- | --- |
| WBC per mm3 | 3970 | 5200-1100 | 6010 | 4400-9500 | 5790 | 440-8100 |
| Lymphocyte (Absolute count) | 871 | 2300-5400 | 3360 | 1900-3700 | 2670 | 1400-3300 |
| CD3+ %Lys | 50% (435) | 56-75% (1400-3700) | 42% (1411) | 60-76% (1200-2600) | 31% (828) | 56-84% (1000-2200) |
| CD4+ %Lys | 13,4% (116) | 28-47% (700-2700) | 19% (638) | 31-47% (650-1500) | 14% (374) | 31-52% (530-1300) |
| CD8+%Lys | 21,7% (189) | 16-30% (490-1300) | 11% (369) | 18-35%  (370-1100) | 10% (267) | 18-35% (330-920) |
| CD16+CD56 +%Lys | 19,5% (170) | 4-17% (130-720) | 5% (168) | 4-17%  (100-480) | 3%  (80) | 3-22% (70-480) |
| CD19+ %Lys | 26% (226) | 14-33% (390-1400) | 44% (1474) | 13-27%  (270-860) | 66%  (1762) | 6-23% (110-570) |
| CD4+ CD45RA+ (naive) on % CD4+ | 9,6% (11) | 53-86% (430-1500) | 19% (121) | 46-77% (320-1000) | 10% (37) | 33-66% (230-770) |
| CD8+CD45RA + (naive)  on % CD8+ | 29% (55) | 69-97% (380-1100) | 7% (26) | 63-92%  (310-900) | 6% (16) | 61-91% (240-710) |
| Serum immunoglobulin levels (mg/dl) | Pt  (5-years-old) | Normal values for age[S10] | Pt (7-years-old) | Normal values for age [S10] | Pt (13-years-old) | Normal values for age [ S10] |
| IgG | 453 | 528-1959 | 1152^+^ | 633-1016 | 923^+.^ | 604-1909 |
| IgA | 102 | 37-257 | 27 | 41-315 | 33 | 61-301 |
| IgM | 63 | 49-249 | 19 | 56-261 | 21 | 59-297 |
|  |  |  |  |  |  |  |
| Blood group isohemagglutinins (IgM–anti-B) | 1:4 | (>1:8) | 1:4 | (>1:8) | nd | (>1:8) |
|  |  |  |  |  |  |  |
| B cell subsets  % on CD19+ | Pt  (5-years-old) | % Normal values for age[S11] | Pt (7-years-old) | %Normal values for age [ S11] | Pt (13-years-old) | %Normal values for age [S11] |
| CD19+CD27+ (memory) | 453 | 14,7 %(7,0-24) | 1.43% | 18.4% (8.1–33.3) | 2.0% | 16.0% (7.0-29.0) |
| CD27+IgD+ | 0,78% | 7,2%(4,6-16,3) | 0,77% | 7.3% (3.1–18.0) | nd | 6.4% (2.6–13.4) |
| CD27+ IgD- | 0,36% | 6,9%(2,7-12,5) | 0.67% | 8.4 %(2.9–17.4) | nd | 9.1% (4.0–21.2) |
| CpG stimulation | nd |  | nd |  | Low level of IgA and IgM;absent production of IgG |  |
| WBC per mm3 | 3970 | 5200-1100 | 6010 | 4400-9500 | 5790 | 440-8100 |

Patient data have been on follow-up in Immuno-Infectivology Unit of the Pediatric Hospital Bambino Gesù. Brackets indicate absolute numbers. Normal range for age reported in reference S9-11. ^+^Patient on IVIG supplementation. Other normal values were measured according to internal laboratory references. nd: not determined.

**Table S2. Exome sequencing analysis**

| PT | Total number of reads | 113.469.575 |
| --- | --- | --- |
|  | Coverage | 109 |
|  | All rare variants | 110 |
|  | All Heterozygous | 97 |
|  | Heterozygous non synonymous | 77 |
|  | Heterozygous indels | 9 |
|  | Heterozygous frameshift | 6 |
|  | Heterozygous splice site | 4 |
|  | Heterozygous stop gained | 2 |
|  | Heterozygous exon | - |
|  | All Homozygous | 13 |
|  | Homozygous  non synonymous | 1 |
|  | Homozygous indels | 4 |
|  | Homozygous frameshift | 5 |
|  | Homozygous splice site | - |
|  | Homozygous stop gained | - |
|  | Homozygous exon | 2 |

**Table S3. Primers for DNA analysis**

| Region | Orientation | Sequence | PCR CYCLES |
| --- | --- | --- | --- |
| 1 | FOR | CGCAGGCTGGTCTTAAACTC | 94°C 3 min, 94°C 30 sec- 58°C 30 sec- 72°C 1 min X 40 cycles, 72°C 10 min.  94°C 3 min, 94°C 30 sec- 63.4°C 30 sec- 72°C 1 min X 40 cycles, 72°C 10 min. |
|  | REV | CTGAAGTGCACAGGTGAAATT |  |
| 2 | FOR | TATGTTTGTTGGCCGCATAA |  |
|  | REV | AGAAGGGAACCTCCTCAACC |  |
| Combination | 1REV | CTGAAGTGCACAGGTGAAATT | 94°C 3 min, 94°C 30 sec- 61°C 30 sec- 72°C 2 min X 40 cycles, 72°C 10 min. |
|  | 2FOR | TATGTTTGTTGGCCGCATAA |  |

**Table S4. Primers for cDNA analysis**

| **cDNA sequencing by PCR** | | | | | | | |
| --- | --- | --- | --- | --- | --- | --- | --- |
| Region | Orientation | | | Sequence | PCR CYCLES | | Bp size |
| 8 | FOR | | | TATGTTTGTTGGCCGCATAA | 94°C 3 min, 94°C 30 sec- 58.1°C 30 sec- 72°C 45 sec X 45 cycles, 72°C 10 min. | | 944bp |
| 1 | REV | | | GAAGGGAGAGGAGCGAACAT |  |  |  |
| 2 | FOR | | | GCAGTTGGAGAGCAGTGAAC | 94°C 3 min, 94°C 30 sec- 54.9°C 30 sec- 72°C 1 min X 40 cycles, 72°C 10 min. | | 268bp |
| 1 | REV | | | GAAGGGAGAGGAGCGAACAT |  |  |  |
| **mRNA analysis by quantitative RT-PCR** | | | | | | | |
| MAGT1 | | FOR | TGCTACCTCTGACATGGATATTG | | | 50° 2min, 95° 15min, 95° 15 sec-60° 1min x 40 cycles | |
|  |  | REV | AGAGAGCATCCAACTGAAGAATAA | | |  |  |
|  |  | PROBE | FAM-TGTGTGTGGCTGGTATTGGACTTGT | | |  |  |
| TELO | | FOR | GGCACACGTGGCTTTTCG | | |  |  |
|  |  | REV | GGTGAACCTCGTAAGTTTATGCAA | | |  |  |
|  |  | PROBE | VIC-TCAGGACGTCGAGTGGACACGGTG | | |  |  |
